# Derived Neutrophil-to-Lymphocyte Ratio Predicts Pathological Complete Response to Neoadjuvant Chemotherapy in Breast Cancer

**DOI:** 10.3389/fonc.2021.827625

**Published:** 2022-02-11

**Authors:** Alberto Ocaña, Jose Ignacio Chacón, Lourdes Calvo, Antonio Antón, Mauro Mansutti, Joan Albanell, María Teresa Martínez, Ainhara Lahuerta, Giancarlo Bisagni, Begoña Bermejo, Vladimir Semiglazov, Marc Thill, Arlene Chan, Serafin Morales, Jesús Herranz, Ignacio Tusquets, Massimo Chiesa, Rosalía Caballero, Pinuccia Valagussa, Giampaolo Bianchini, Emilio Alba, Luca Gianni

**Affiliations:** ^1^ Hospital Clínico San Carlos, Madrid e Instituto de Investigación Sanitaria San Carlos (IdISSC), Madrid and Universidad de Castilla La Mancha, Albacete, Spain; ^2^ Centro de Investigación Biomédica en Red de Oncología, CIBERONC-ISCIII, Madrid, Spain; ^3^ GEICAM Spanish Breast Cancer Group, San Sebastián de los Reyes, Madrid, Spain; ^4^ Oncology Department, Hospital Virgen de la Salud, Toledo, Spain; ^5^ Oncology Department, Complejo Hospitalario Universitario de A Coruňa, A Coruňa, Spain; ^6^ Hospital Universitario Miguel Servet, Instituto de Investigación Sanitaria de Aragón (IISA), Zaragoza, Spain; ^7^ Oncology Department, University Hospital, Udine, Italy; ^8^ Cancer Research Program, Hospital del Mar Medical Research Institute (IMIM), Oncology Department, Hospital del Mar, Barcelona, Spain; ^9^ Universitat Pompeu Fabra, Barcelona, Spain; ^10^ Hospital Clínico Universitario de Valencia, Biomedical Research Institute INCLIVA, Valencia, Spain; ^11^ Oncology Department, Onkologikoa, San Sebastián, Spain; ^12^ Oncology Department, Azienda USL-IRCCS di Reggio Emilia, Reggio Emilia, Italy; ^13^ Oncology Department, NN Petrov Research Inst of Oncology, St. Petersburg, Russia; ^14^ Oncology Department, Agaplesion Markus Krankenhaus, Frankfurt am Main, Germany; ^15^ Breast Cancer Research Center, Curtin University, Perth, WA, Australia; ^16^ Oncology Department, Hospital Universitario Arnau de Vilanova de Lleida, Lleida, Spain; ^17^ Fondazione Michelangelo, Milano, Italy; ^18^ Hospitales Universitarios Regional y Virgen de la Victoria, IBIMA, Malaga, Spain

**Keywords:** breast cancer, neoadjuvant chemotherapy, DNLR, PCR, immunology

## Abstract

**Background:**

Derived neutrophil-to-lymphocyte ratio (dNLR) is a biomarker associated with clinical outcome in breast cancer (BC). We analyzed the association of dNLR with pathological complete response (pCR) in triple-negative BC (TNBC) patients receiving neoadjuvant chemotherapy (CT).

**Methods:**

This is a retrospective analysis of two randomized studies involving early stage/locally advanced TNBC patients receiving anthracycline/taxane-based CT+/−carboplatin (GEICAM/2006-03) or nab-paclitaxel/paclitaxel followed by anthracycline regimen (ETNA). dNLR was calculated as the ratio of neutrophils to the difference between total leukocytes and neutrophils in peripheral blood before CT (baseline) and at the end of treatment (EOT). Logistic regression analyses were used to explore dNLR association with pCR.

**Results:**

In total, 308 TNBC patients were analyzed, 216 from ETNA and 92 from GEICAM/2006-03. Baseline median dNLR was 1.61 (interquartile range (IQR): 1.25–2.04) and at EOT 1.53 (IQR: 0.96–2.22). Baseline dNLR showed positive correlation with increased tumor size (*p*-value = 1e−04). High baseline dNLR, as continuous variable or using median cutoff, was associated with lower likelihood of pCR in univariate analysis. High EOT dNLR as continuous variable or using quartiles was also associated with lower pCR rate in uni- and multivariate analyses.

**Conclusions:**

High baseline and EOT dNLR correlates with lower benefit from neoadjuvant CT in TNBC.

## Introduction

Neoadjuvant chemotherapy is a widely used therapeutic option for the treatment of early-stage or locally advanced breast cancer (1). This is particularly evident for the treatment of HER2-positive breast cancer or the triple-negative breast cancer (TNBC) subtypes in which pathological complete response (pCR) has been shown to be associated with improved clinical outcome (2–5). Further, patients with TNBC or HER2 positive tumors that do not achieve a pCR can be offered additional adjuvant treatment including TDM1 or chemotherapy, respectively, which has demonstrated an improvement in survival (5, 6). For TNBC, although not all clinical studies have confirmed this benefit, residual invasive disease following neoadjuvant chemotherapy is indicative of high risk of relapse and additional adjuvant treatment with capecitabine can contribute to the reduction of this risk (7, 8). Unfortunately, the benefit of neoadjuvant chemotherapy in other subtypes of breast cancer, particularly in the luminal group, is less clear and is restricted to locally advanced cases.

Inflammation as a consequence of the immune response to the tumor is a hallmark of cancer (9). In addition, the presence of an immunogenic activated environment identifies tumors that have a better prognosis and predicts for response to immune checkpoint inhibitors (ICIs) (10, 11). TNBC demonstrates heightened immunogenic activation, but data with ICIs in the advanced disease have demonstrated disappointing results, with only one study showing an increase in overall survival when the population was selected by PD-L1 expression (12). In one study, pembrolizumab in combination with chemotherapy in the neoadjuvant setting slightly reached the prespecified pathological complete response threshold to consider the experimental arm as being beneficial (13) The ability to identify biomarkers that can help to select patients whose tumors are most likely to respond to ICIs in combination with chemotherapy would be clinically important. Although it is known that immunologic transcriptomic signatures can identify immune-active tumors that can better respond to chemotherapy (14), the evaluation of biomarkers which can be more easily implemented is a clinical necessity.

The derived neutrophil-to-lymphocyte ratio (dNLR) is calculated as the ratio of neutrophils to the difference between total leukocytes and neutrophils in peripheral blood (15). Its role to discriminate prognosis has been widely explored and in some indications has already been incorporated in clinical guidelines (e.g., prostate cancer).

It is clear that the use of liquid biopsy to study genomic correlates of the tumor or to indirectly evaluate biomarkers of immune response has gained momentum, demonstrating its utility in different clinical scenarios (16).

Our group and others have explored the role of dNLR in early-stage breast cancer demonstrating its prognostic value (17–19). The role of dNLR in the neoadjuvant setting to predict response to chemotherapy has not been established.

To this end, we evaluated two randomized studies (GEICAM/2006-03-NCT00432172 and ETNA-NCT01822314) which investigated neoadjuvant chemotherapy in operable early stage (>2 cm; node positive) or locally advanced tumors to explore the capability of dNLR to predict response in the TNBC subgroup.

## Material and Methods

### Clinical Trials and Patients

Data from patients who participated in the randomized phase 2 GEICAM/2006-03 (NCT00432172) and phase 3 ETNA (NCT01822314) trials were analyzed retrospectively. Details and main results of the studies were published elsewhere (20–22). Briefly, in the GEICAM/2006-03 trial, HER2-negative patients were selectively treated according to clinical subtypes: triple-negative (TN) patients received standard taxane/anthracycline-based chemotherapy (TA-CT) +/− carboplatin, while luminal patients were randomized to TA-CT vs. hormone therapy (only patients (pts) that received TA-CT +/− carboplatin were considered for this analysis). In the ETNA trial, HER2-negative pts were treated with nab-paclitaxel or paclitaxel followed by anthracyclines. In both studies, pCR in breast and axilla was used to measure treatment response according to Miller&Payne criteria. Analysis of ER, PgR, and HER2 status was carried out in a central laboratory in the two studies. TN subgroup was defined as estrogen receptor (ER) negative, progesterone receptor (PgR) negative, and HER2.

### dNLR Calculation

dNLR was calculated from analytical values of peripheral blood collected either before the start of chemotherapy (baseline) or at the end of treatment (EOT). dNLR was calculated as the ratio of the absolute neutrophil number to the difference between absolute total leukocyte and absolute neutrophil counts, a proxy for lymphocyte count (22). Patients without information on neutrophils and leukocytes and patients with leukocyte counts >15 × 109/L were excluded, as this might reflect infectious or hematologic conditions unrelated to breast cancer.

### Statistical Analyses

Univariate and multivariate logistic regression analyses were used to explore the association of dNLR with main clinical characteristics and dNLR capability (distributed as a continuous variable, using median cutoff and quartiles) to predict pCR. Multivariate models were adjusted for important clinical variables (treatment, tumor size, lymph nodes, grade, Ki67) and for clinical variables significantly associated (*p*-value <0.1) with pCR (age, histological type). An optimal cutoff model based on Youden Index was also used to analyze the association of basal dNLR with pCR.

## Results

### Clinical and Pathological Patients’ Characteristics

A total of 308 patients with TNBC were included in the analysis, 216 from the ETNA and 92 from the GEICAM/2006-03 study. Both trials evaluated neoadjuvant chemotherapy in early stage (tumor >2 cm and/or node positive) or locally advanced triple-negative and luminal breast cancers. Median age of the analyzed patients was 51 years. Most patients had cT2 disease (*n* = 227, 73.7%)and cN1 (*n* = 153, 49.7%) followed by cN0 (*n* = 133, 43.2%). pCR was achieved in 36.7% (*n* = 113) of the treated patients. All patients’ characteristics, according to response to chemotherapy (pCR = No/Yes) are described in **Table 1**.

**Table 1:** Main clinical-pathological patients’ characteristics.

Variable	All patients (*N* = 308; 100%)	ETNA patients (*N* = 216; 70.1%)	GEICAM/2006-03 patients (*N* = 92; 29.9%)	pCR = No (*N* = 195; 63.3%)	pCR = Yes (*N* = 113; 36.7%)
Tumor size					
cT1	9 (2.9%)	0 (0%)	9 (9.8%)	7 (3.6%)	2 (1.8%)
cT2	227 (73.7%)	164 (75.9%)	63 (68.5%)	134 (68.7%)	93 (82.3%)
cT3	51 (16.6%)	33 (15.3%)	18 (19.5%)	38 (19.5%)	13 (11.5%)
cT4	19 (6.2%)	17 (7.9%)	2 (2.2%)	15 (7.7%)	4 (3.6%)
NA	2 (0.7%)	2 (0.9%)	0 (0%)	1 (0.5%)	1 (0.9%)
Lymph nodes					
cN0	133 (43.18%)	128 (59.2%)	5 (5.4%)	80 (41.03%)	53 (46.9%)
cN1	153 (49.68%)	68 (31.5%)	85 (92.4%)	105 (53.85%)	48 (42.48%)
cN2	20 (6.49%)	20 (9.3%)	0 (0%)	9 (4.62%)	11 (9.73%)
NA	2 (0.65%)	0 (0%)	2 (2.2%)	1 (0.51%)	1 (0.88%)
Tumor grade					
G1	3 (1.0%)	0 (0%)	3 (3.3%)	2 (1.0%)	1 (0.9%)
G2	46 (15.0%)	25 (11.6%)	21 (22.8%)	40 (20.5%)	6 (5.3%)
G3	133 (43.2%)	65 (30.1%)	68 (73.9%)	106 (54.4%)	27 (23.9%)
NA	126 (40.8%)	126 (58.3%)	0 (0%)	47 (24.1%)	79 (69.9%)
Ki67 14%					
Low (<14%)	5 (1.6%)	3 (1.4%)	2 (2.2%)	4 (2.1%)	1 (0.9%)
High (≥14%)	286 (92.9%)	213 (98.6%)	73 (79.3%)	176 (90.2%)	110 (97.4%)
NA	17 (5.5%)	0 (0%)	17 (18.5%)	15 (7.7%)	2 (1.8%)
Ki67 21%					
Low (<21%)					
High (≥21%)					
NA					

pCR, pathological complete response; NA, not available.

### dNLR Expression and Association With Clinical Characteristics

**Figure 1:**
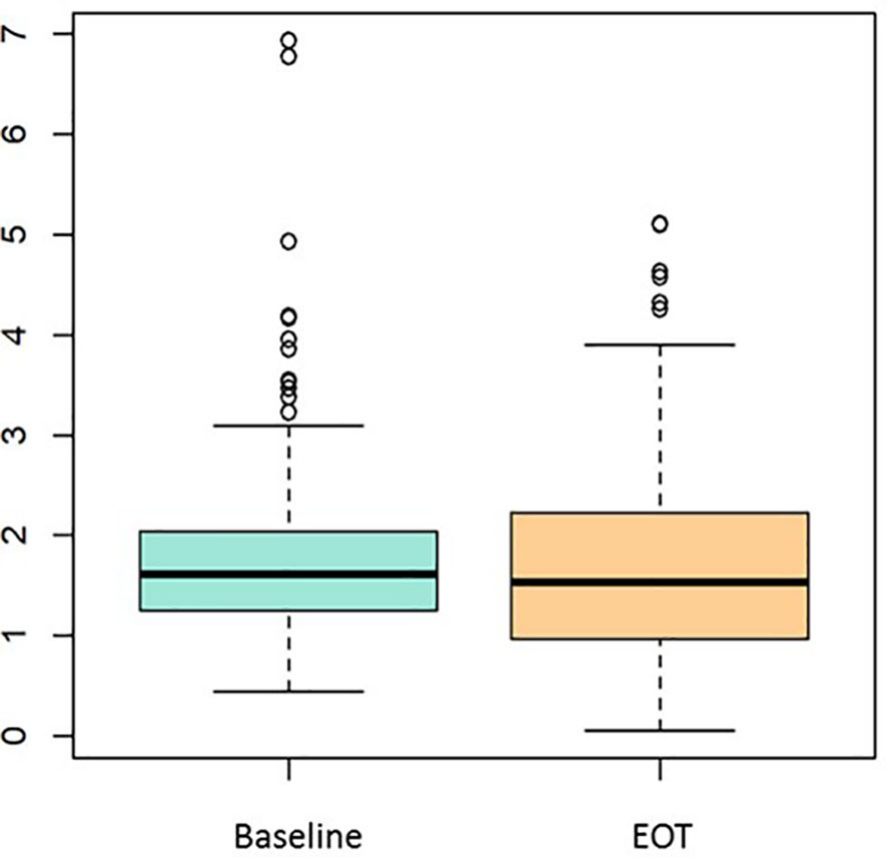
dNLR median values before CT (Baseline) and at EOT. dNLR, derived neutrophil-to-lymphocyte ratio; CT, chemotherapy; EOT, end of treatment.

Median baseline dNLR was 1.61 (IQR: 1.25–2.04), and at the EOT 1.53 (IQR: 0.96–2.22) (**Figure 1**) (**Table 2**).

**Table 2:** dNLR expression in the patients’ population.

	TNBC
	*n* = 308 (37.5%)
dNLR baseline	
*N*	308
Median	1.61
Lower quartile	1.25
Upper quartile	2.04
dNLR EOT	
*N*	255
Median	1.53
Lower quartile	0.96
Upper quartile	2.22

dNLR, derived neutrophil-to-lymphocyte ratio; TNBC, triple-negative breast cancer; EOT, end of treatment.

Analysis of the association of baseline dNLR with the main clinical features (tumor size, lymph nodes, tumor grade, Ki67) demonstrated a significant correlation only with tumor size (*p*-value = 1e−04) (**Table 3**). No significant associations were found between EOT dNLR and clinical features (**Table 3**).

**Table 3:** Association of dNLR with clinical and pathological parameters in TNBC.

Variable	Category	*N*	Mean (SD)	Rho^a^	*p*-value^b^
dNLR baseline					
Tumor size	cT1	9	1.252 (0.45)		0.0001
	cT2	227	1.685 (0.756)		
	cT3	51	2.002 (0.864)		
	cT4	19	1.943 (0.73)		
	NA	2	0.965 (0.077)		
Lymph nodes	cN0	133	1.731 (0.927)		0.1726
	cN1	153	1.708 (0.603)		
	cN2	20	2.011 (0.882)		
Tumor grade	G1	3	1.832 (0.395)		0.3981
	G2	46	1.689 (0.571)		
	G3	133	1.772 (0.828)		
	NA	100	1.632 (0.635)		
Ki67		291		0.049	0.4049^*^
dNLR EOT					
Tumor size	cT1	6	1.759 (0.927)		0.6763
	cT2	189	1.653 (0.968)		
	cT3	43	1.58 (1.064)		
	cT4	15	1.858 (1.031)		
	NA	2	1.028 (0.519)		
Lymph nodes	cN0	118	1.533 (0.945)		0.0613
	cN1	120	1.794 (1.01)		
	cN2	16	1.441 (0.973)		
Tumor grade	G1	2	1.801 (1.124)		0.0938
	G2	36	1.815 (1.174)		
	G3	108	1.771 (1.002)		
	NA	95	1.445 (0.883)		
Ki67		242		−0.001	0.9866^*^

dNLR, derived neutrophil-to-lymphocyte ratio; TNBC, triple-negative breast cancer; SD, standard deviation; EOT, end of treatment.

a Spearman correlation.

b Mann-Whitney/Kruskal-Wallis/Correlation test.

^*^Spearman’s p-value.

No association was observed between baseline dNLR and EOT dNLR (correlation coefficient: 0.138).

### Association of Baseline and EOT dNLR With pCR

In the univariate analysis, high baseline dNLR level, considered continuous and categorical variable defined by the median cutoff, was associated with a lower likelihood of pCR (OR: 0.709; 95% CI: 0.5–1.006, *p*-value = 0.0406 and OR: 0.59; 95% CI: 0.37–0.94, *p*-value = 0.0244, respectively). The association between baseline dNLR quartiles and pCR was only marginally significant (*p*-value = 0.0706). The association did not retain significance in multivariate analysis after correction for clinical variables (**Table 4**).

**Table 4:** Association of baseline and EOT dNLR with pCR in TNBC patients’ population.

dNLR distribution	Category	Baseline dNLR					EOT dNLR				
		pCR = yes (%)	*Univariate analysis*		*Multivariate analysis*		pCR = yes (%)	*Univariate analysis*		*Multivariate analysis*	
			OR (CI 95%)	*p*-value^*^	OR (CI 95%)	*p*-value^*^		OR (CI 95%)	*p*-value^*^	OR (CI 95%)	*p*-value^*^
dNLR continuous			0.709 (0.5–1.006)	0.0406	0.691 (0.418–1.14)	0.14		0.665 (0.501–0.884)	0.0034	0.62 (0.406–0.946)	0.0231
dNLR median	Low dNLR	42.9	1	0.0244	1	0.1105	43.3	1	0.084	1	0.1248
	High dNLR	30.5	0.59 (0.37–0.94)		0.59 (0.31–1.13)		32.8	0.64 (0.38–1.06)		0.56 (0.27–1.18)	
dNLR quartiles	Q1	48.1	1	0.0706	1	0.3652	45.3	1	0.006	1	0.021
	Q2	37.7	0.65 (0.34–1.24)		0.82 (0.34–2)		41.3	0.85 (0.42–1.71)		0.92 (0.32–2.6)	
	Q3	32.5	0.52 (0.27–1)		0.61 (0.24–1.55)		45.3	1 (0.5–2.01)		1.12 (0.39–3.21)	
	Q4	28.6	0.43 (0.22–0.84)		0.45 (0.17–1.16)		20.3	0.31 (0.14–0.67)		0.26 (0.09–0.75)	
dNLR 3Q	1–2–3Q	39.4	1	0.0839	1	0.1488	44.0	1	0.0005	1	0.002
	4Q	28.6	0.62 (0.35–1.08)		0.57 (0.26–1.23)		20.3	0.32 (0.17–0.64)		0.26 (0.1–0.63)	

p-values set in bold indicate statistical significance.

EOT, end of treatment; dNLR, derived neutrophil-to-lymphocyte ratio; pCR, pathological complete response; TNBC, triple-negative breast cancer; Q, quartile; OR, odds ratio; CI, confidence interval.

^*^Likelihood ratio test (LRT).

Bold p-values are statistically significant values.

High EOT dNLR levels considered a continuous variable was associated with lower likelihood of achieving a pCR, in both univariate (OR: 0.665; 95% CI: 0.501–0.884; *p*-value = 0.0034) and multivariate (OR: 0.62; 95% CI: 0.406–0.946; *p*-value = 0.0231) logistic regression analysis. High EOT dNLR defined by the median cutoff demonstrated only a trend for association with lower pCR (OR: 0.64; 95% CI: 0.38–1.06; *p*-value = 0.084). When EOT dNLR was assessed by quartiles, a significant association between higher dNLR and lower rate of pCR was described in both uni- and multivariate analyses (*p*-value = 0.006 and 0.021, respectively). This association was driven by the highest quartile as demonstrated by the comparison with the combined lower quartiles (OR: 0.32; 95% CI: 0.17–0.64; *p*-value = 5e−04 and OR: 0.26; 95% CI: 0.1–0.63; *p*-value = 0.002, respectively).

An exploratory assessment of dNLR to define the optimal predictive cutoff points was performed: baseline cutoff = 1.715 (OR: 0.65; 95% CI: 0.51–0.82; *p*-value = 0.035) and EOT dNLR cutoff = 2.231 (OR: 0.43; 95% CI: 0.25–0.74; *p*-value = 0.0035) (**Table 5**).

**Table 5:** Association of baseline and EOT dNLR with pCR in TNBC patients’ population—cutpoint model.

Variable	Categ^a^	*N*	OR (95% CI)^b^	*p*-value^c^
dNLR baseline	<1.715	182	1	0.035
	≥1.715	126	0.65 (0.51–0.82)	
dNLR EOT	<2.231	192	1	0.0035
	≥2.231	63	0.43 (0.25–0.74)	

EOT, end of treatment; dNLR, derived neutrophil-to-lymphocyte ratio; pCR, pathological complete response; TNBC, triple-negative breast cancer; OR, odds ratio; CI, confidence interval.

a Selected cutpoint maximizing the statistic of the likelihood ratio test in the logistic regression: 1.715. Cutpoint maximizing the Youden Index from the ROC curve: 1.715.

b Estimated effect based in a twofold cross-validation (D Faraggi and R Simon).

c p-value based in a permutation test (S G Hilsenbeck and G M Clark).

**Supplementary Tables S1–S3** describe the variables included in the multivariate logistic regression analysis.

## Discussion

We described a new biomarker related to inflammation and immune response which is associated with a lower likelihood to achieve pCR in patients with TNBC treated with chemotherapy in these two prospective randomized trials. Identification of patients who derive the greatest benefit from chemotherapy is useful to optimize treatment tailoring and therefore the discovery of novel predictive biomarkers that could be easily implemented in the clinic is warranted.

Achieving a pCR has been associated with better clinical outcome particularly for the triple-negative and HER2-positve subtypes (23).

TNBC is the more immune-activated breast cancer subtype, and incorporation of immune checkpoint inhibitors in combination with chemotherapy in high-risk TNBC appears promising and will likely become the new standard of care (24, 25). However, the current unmet need is the precise identification of patients who will most likely benefit from the addition of immune checkpoint therapy to the standard of care.

In the case of TNBC, predictive biomarkers of response to neoadjuvant chemotherapy have been demonstrated both in terms of tumor-infiltrating lymphocytes and transcriptomic signatures (26–28). In our study, dNLR appeared to be another promising easily assessable and reliable biomarker that could be incorporated into the clinical practice and might help to identify those patients with lower benefit from chemotherapy and who might benefit the most from additional treatments.

How chemotherapy can affect inflammation in TN subtype is unclear, but some studies have suggested that it could modulate immune populations therefore influencing treatment efficacy (29). Conversely, systemic inflammation could be associated with different local immune milieu such as the amount of tumor-infiltrating lymphocytes, which are known to influence prognosis and response to chemotherapy.

In this study, no association between dNLR and clinicopathological parameters was observed with the exception of larger tumor size which was associated with high dNLR. To this regard, the association with size can just confirm the more aggressive phenotype recognized by the presence of dNLR.

EOT dNLR as a continuous variable and by quartile distribution was associated with a reduce rate of pCR in TNBC in univariate and multivariate analyses. This finding suggests that administration of chemotherapy may affect the immune system within the tumor, inducing inflammation that somehow could limit the efficacy of the chemotherapy (29). Alternatively, it is known that chemotherapy can stimulate an immunologic cell death, which depends on the tumor type and the specific genomic and stromal microenvironment and could favor the efficacy of chemotherapy (30).

Assessment of dNLR in TNBC using the optimal cutoff showed a negative association with pCR in the Cox regression model at baseline and at the EOT dNLR. This suggests that high baseline dNLR may also be informative as to which patients are less likely to benefit from chemotherapy. Assessment of this biomarker before treatment can be implemented easily, thus potentially allowing its incorporation into management decisions. In this context, integration of liquid biopsy to help the management of our patients is gaining momentum, and the reported analysis here is an example of one of those applications.

We recognize that there are limitations in our analysis. Firstly, this is a retrospective analysis which would need to be confirmed ideally in a prospective clinical trial. Secondly, it is unclear if the observed association of dNLR levels and lower likelihood to response to chemotherapy would be influenced when ICIs are used together with chemotherapy. In this regard, the analysis of dNLR in patients treated with ICIs in combination with chemotherapy in the neoadjuvant setting warrants investigation, as it might identify a subgroup of patients who derive less benefit also from ICIs.

High levels of dNLR correlate with lower likelihood to benefit from chemotherapy in TN tumors receiving neoadjuvant chemotherapy. This finding warrants confirmation in larger retrospective or prospective cohort of patients and in the context of inclusion of ICIs use in the neoadjuvant setting.

## Conclusions

Our results suggest that high dNLR levels at baseline and especially at EOT are associated with lower likelihood of achieving a pCR in patients with TNBC treated with neoadjuvant chemotherapy. The reported data should be considered exploratory, and the evaluation in an additional dataset will be necessary to confirm these results. Similarly, future studies should evaluate the role of dNLR to predict response of ICIs in combination with chemotherapy.

## Data Availability Statement

The raw data supporting the conclusions of this article will be made available by the authors, without undue reservation.

## Ethics Statement

The studies involving human participants were reviewed and approved by the Institut Municipal d’Assistencia Sanitaria (IMAS), Paseo Marítimo 25-29, 08003 Barcelona, Parc de Salut Mar, Dr. Aiguader 88, 08003 Barcelona. The patients/participants provided their written informed consent to participate in this study.

## Author Contributions

Conceptualization: AO, EA, and LG. Methodology: AO and JH. Software: JH, MC, and RC. Validation and formal analysis: JH. Investigation and data curation: AO, JC, LC, AA, MM, MTM, JA, AL, GBis, BB, VS, MT, AC, SM, IT, PV, GBia, EA, and LG. Resources: AO, JC, LC, AA, MM, MTM, JA, AL, GBis, BB, VS, MT, AC, SM, IT, PV, GBia, EA, LG, MC, and RC. Writing—original draft preparation: AO, GBia, EA, MC, and RC. Writing—review and editing and visualization: all authors. Supervision: AO, MC, and RC. Project administration: MC and RC. Funding acquisition: AO.

## Funding

This analysis was supported by GEICAM Spanish Breast Cancer Group.

## Conflict of Interest

AO is currently an employee of Symphogen, Denmark. MM has received honoraria from Pierre Fabre and support for attending meetings and/or travel from Eisai, Novartis, Pfizer, Pierre Fabre, and Roche. He has received advisory board honoraria from Amgen, Astra Zeneca, Eli Lilly, Gentili, MSD Italia, Novartis, Pfizer, and Roche. JA has received advisory board honoraria from Roche, Lilly, Merck, Daiichi-Sankyo/Astrazeneca, and Seagen and a speaker’s honoraria from Pfizer. GBia has received honoraria from Pfizer, Roche, AstraZeneca, Lilly, Novartis, Noepharm Israel, Amgen, MSD, Chugai, Sanofi, Daiichi Sankyo, Eisai, Gilead, Seagen, and Exact Science. EA has received advisory board honoraria from Roche, Novartis, Pfizer, Lilly, BMS, Genomic Health, and Nanostring and support for attending meetings and/or travel from Celgene, as well as investigation grants from Roche, Pfizer, Sysmex, MSD, and Nanostring. LG has received advisory board honoraria from ADC Therapeutics, AstraZeneca, Celgene, Eli Lilly, G1 Therapeutics, Genentech, Genomic Health, Merck Sharp & Dohme, Oncolytics Biotech, Odonate Therapeutics, Onkaido Therapeutics, Roche, Pfizer, Taiho Pharmaceutical, Hexal Sandoz, Seattle Genetics, Synthon, Zymeworks, and Sanofi-Aventis; consultant honoraria for selected programs of Forty Seven (CD47), GENENTA, METIS Precision Medicine, Novartis, Odonate Therapeutics, Revolution Medicines, Synaffix, Zymeworks, Menarini Ricerche, Amgen, and Biomedical Insights; and research support from Zymeworks, Revolution Medicines (his institution). LG is coinventor of “European Patent Application N.12195182.6 and 12196177.5,” titled “PDL-1 expression in anti-HER2 therapy”—Roche (no compensation provided).

The remaining authors declare that the research was conducted in the absence of any commercial or financial relationships that could be construed as a potential conflict of interest.

## Publisher’s Note

All claims expressed in this article are solely those of the authors and do not necessarily represent those of their affiliated organizations, or those of the publisher, the editors and the reviewers. Any product that may be evaluated in this article, or claim that may be made by its manufacturer, is not guaranteed or endorsed by the publisher.
