# Supplementary material for: Derived Neutrophil-to-Lymphocyte Ratio Predicts Pathological Complete Response to Neoadjuvant Chemotherapy in Breast Cancer
Source: Front Oncol. 2022 Feb 11;11:827625. doi: 10.3389/fonc.2021.827625 (PMC8875201; doi:10.3389/fonc.2021.827625)
Supplement: Supplementary file 1 [file Table_1.docx]

Supplementary table 1. Association between pCR and EOT dNLR Continuous and clinical variables using multivariate logistic regression analysis

| **Variable** | **Categ** | **OR(CI95%)** | **p-value(1)** |
| --- | --- | --- | --- |
| EOT dNLR Continuous |  | 0.62 (0.406-0.946) | 0.0231 |
| Tumor size | T1 | 1 | 0.143 |
|  | T2 | 3.419 (0.333-35.108) |  |
|  | T3 | 1.033 (0.09-11.924) |  |
|  | T4 | 1.671 (0.09-30.929) |  |
|  | Other | 3.154 (0.011-871.231) |  |
| Lymph nodes | N0 | 1 | 0.797 |
|  | N1 | 0.942 (0.388-2.287) |  |
|  | N2 | 1.676 (0.313-8.981) |  |
| Grade | G1 | 1 | <0.0001 |
|  | G2 | 0.351 (0.014-8.969) |  |
|  | G3 | 0.194 (0.008-4.931) |  |
|  | GX | 9.603 (0.356-258.755) |  |
| Ki67(cont.) |  | 1.018 (0.997-1.039) | 0.083 |
| Treatment arm | Nab-P -> AC/EC or FEC | 1 | 0.0463 |
|  | P -> AC/EC or FEC | 1.267 (0.332-4.837) |  |
|  | Nab-P | 0.193 (0.038-0.98) |  |
|  | P | 0.19 (0.04-0.907) |  |

Supplementary table 2. Association between pCR and EOT dNLR by dNLR quartiles and clinical variables using multivariate logistic regression analysis

| **Variable** | **Categ** | **OR(CI95%)** | **p-value(1)** |
| --- | --- | --- | --- |
| EOT dNLR Quartiles | Q1 | 1 | 0.021 |
|  | Q2 | 0.917 (0.323-2.6) |  |
|  | Q3 | 1.12 (0.39-3.212) |  |
|  | Q4 | 0.259 (0.089-0.754) |  |
| Tumor size | T1 | 1 | 0.2224 |
|  | T2 | 2.681 (0.244-29.434) |  |
|  | T3 | 0.914 (0.072-11.529) |  |
|  | T4 | 0.93 (0.044-19.725) |  |
|  | Other | 2.1 (0.007-662.095) |  |
| Lymph nodes | N0 | 1 | 0.5708 |
|  | N1 | 0.993 (0.405-2.432) |  |
|  | N2 | 2.418 (0.427-13.691) |  |
| Grade | G1 | 1 | <0.0001 |
|  | G2 | 0.19 (0.006-5.611) |  |
|  | G3 | 0.113 (0.004-3.227) |  |
|  | GX | 5.483 (0.183-164.348) |  |
| Ki67(cont.) |  | 1.018 (0.997-1.04) | 0.0834 |
| Treatment arm | Nab-P -> AC/EC or FEC | 1 | 0.1458 |
|  | P -> AC/EC or FEC | 1.278 (0.325-5.018) |  |
|  | Nab-P | 0.269 (0.052-1.382) |  |
|  | P | 0.251 (0.052-1.217) |  |

Supplementary table 3. Association between pCR and EOT dNLR (3Q dNR) and clinical variables using multivariate logistic regression analysis

| **Variable** | **Categ** | **OR(CI95%)** | **p-value(1)** |
| --- | --- | --- | --- |
| EOT dNLR 3Q | 1-2-3Q | 1 | 0.002 |
|  | 4Q | 0.257 (0.105-0.631) |  |
| Tumor size | T1 | 1 | 0.2151 |
|  | T2 | 2.905 (0.272-31) |  |
|  | T3 | 0.994 (0.082-11.994) |  |
|  | T4 | 1.057 (0.054-20.802) |  |
|  | Other | 2.201 (0.008-623.025) |  |
| Lymph nodes | N0 | 1 | 0.5788 |
|  | N1 | 1 (0.41-2.437) |  |
|  | N2 | 2.372 (0.429-13.099) |  |
| Grade | G1 | 1 | <0.0001 |
|  | G2 | 0.211 (0.007-6.014) |  |
|  | G3 | 0.123 (0.004-3.45) |  |
|  | GX | 6.061 (0.208-176.899) |  |
| Ki67(cont.) |  | 1.018 (0.997-1.039) | 0.0811 |
| Treatment arm | Nab-P -> AC/EC or FEC | 1 | 0.0895 |
|  | P -> AC/EC or FEC | 1.267 (0.325-4.945) |  |
|  | Nab-P | 0.252 (0.052-1.23) |  |
|  | P | 0.235 (0.051-1.078) |  |
